# Mechanism of Bao Jing Tablets in Chronic Prostatitis/Chronic Pelvic Pain Syndrome: Insights from Multi-Omics and Network Pharmacology

**DOI:** 10.3390/ph19040632

**Published:** 2026-04-17

**Authors:** Haitao Ge, Yan Zhang, Siqi Jin, Chen Wang, Fujiang Wang

**Affiliations:** 1 School of Pharmacy, Nanjing University of Chinese Medicine, Nanjing 210023, China; 2 Jiangsu Suzhong Pharmaceutical Research Institute Co., Ltd., Nanjing 210023, China

**Keywords:** Bao Jing tablet, chronic prostatitis, network pharmacology, metabolomics and proteomics, PI3K/Akt—HIF-1α-Glycolysis pathways

## Abstract

**Background/Objectives**: To investigate the therapeutic potential and mechanistic basis of Bao Jing Tablet (BJT) for chronic prostatitis/chronic pelvic pain syndrome (CP/CPPS) via an experimental autoimmune prostatitis (EAP) rat model, through integrating network pharmacology, metabolomics, proteomics, and animal experiments. **Methods**: UPLC-ZenoTOF 7600-MS/MS was used to analyze the chemical composition of BJT. The therapeutic effect of BJT was evaluated using an experimental autoimmune prostatitis (EAP) rat model. Lipid metabolomics, proteomics, and integrated network pharmacology analyses were performed to investigate the potential mechanisms and active components of BJT in treatment. **Results**: A total of 174 constituents were identified in BJT, among which 54 major active compounds were screened for further analysis. Network pharmacology and combined multi-omics analysis indicate that the protein targets of HIF-1α, Akt, and PI3K/Akt, as well as the Glycolysis pathway, play important roles in the improvement of CP/CPPS. **Conclusions**: Our results demonstrated that BJT was an effective drug to improve the development of CP/CPPS. This is associated with the PI3K/Akt–HIF-1α-Glycolysis pathways.

## 1. Introduction

Chronic prostatitis/chronic pelvic pain syndrome (CP/CPPS) is a clinically common urological disorder with a complex etiology. Its primary characteristics include persistent or recurrent pelvic pain, often accompanied by lower urinary tract symptoms (such as urinary frequency, urgency, and dysuria) and sexual dysfunction [1,2]. Its primary characteristics include persistent or recurrent pelvic pain. Globally, the prevalence of CP/CPPS ranges from approximately 2% to 16%, and in recent years, there has been a trend toward onset at younger ages [3,4]. The pathogenesis of CP/CPPS remains incompletely understood and is generally considered to involve multifactorial interactions, including inflammation, neuroendocrine-immune dysregulation, oxidative stress, and pelvic floor dysfunction [5]. Current clinical management relies primarily on empirical pharmacotherapy, such as alpha-blockers and anti-inflammatory agents; however, these treatments often yield limited efficacy, are associated with side effects, and carry a high risk of recurrence [6,7]. Therefore, exploring novel and effective therapeutic strategies is critical. Traditional Chinese Medicine has shown promising potential in alleviating the clinical symptoms of CP/CPPS, and its multi-component, multi-target mechanisms align well with the complex pathological underpinnings of the disease.

Traditional Chinese Medicine (TCM) posits that CP arises from intrinsic imbalances, encompassing kidney qi deficiency, damp-heat pouring downward, and qi stagnation with blood stasis [8]. BJT is a traditional Chinese medicine compound used to treat prostate diseases. It is formulated based on the TCM principles of “tonifying the kidney, benefiting qi, activating blood circulation, and resolving stasis” [9]. It is derived from two classical prescriptions: Cheng’s Bixie Fen qing formula [10] from Yixue Xinwu and Tusizi Wan [11] from Taiping Huimin Heji Ju Fang. As a hospital preparation (Z04000524) of Jiangsu Provincial Hospital of Traditional Chinese Medicine, BJT consists of ten medicinal ingredients. These ingredients include Schisandrae Chinensis Fructus (*Schisandra chinensis* Baill), Cuscutae Semen (*Cuscuta chinensis* Lam), Dioscoreae Spongiosae Rhizoma (*Dioscorea spongiosa* J.Q.Xi), and Curcumae Rhizoma (*Curcuma phaeocaulis* Val), Poria (*Poria cocos* (Schw.) Wolf), Acori Tatarinowii Rhizoma (*Acorus tatarinowii* Schott), Indigo naturalis (*Baphicacanthus cusia* (Nees) Bremek.), Verbenae herba (*Verbena officinalis* L.), Crassostreae Concha [*Crassostrea gigas* (Thunberg)], Glycyrrhizae Radix Et Rhizoma (*Glycyrrhiza uralensis* Fisch.). In this formula, the “sovereign” herb, Cuscutae Semen, primarily tonifies the kidney and secures essence. Modern pharmacological studies have confirmed that its rich flavonoid components exert clear protective effects on the reproductive system through mechanisms such as hormone regulation, antioxidant activity, and anti-apoptosis [12,13]. Another sovereign herb, Dioscoreae Spongiosae Rhizoma, excels in draining dampness and resolving turbidity. Its core active component, diosgenin, has been shown to possess broad anti-inflammatory and antioxidant properties, exhibiting favorable anti-inflammatory effects in various mouse models of organ injury [14,15,16]. The synergistic combination of all herbs in the formula achieves the dual functions of tonifying the kidney, securing essence, draining dampness, and resolving turbidity, making it particularly suitable for CP characterized by kidney deficiency combined with damp-heat [17]. Although BJT has been clinically applied for decades, and clinical studies have confirmed its efficacy in alleviating symptoms and improving patient quality of life [18,19], its underlying molecular mechanisms have not yet been systematically elucidated. However, existing research has primarily focused on clinical outcomes, and its underlying mechanisms have not yet been systematically elucidated.

The application of omics approaches in the prostate CP/CPPS can help identify novel biomarkers and enhance our understanding of disease mechanisms. Recent studies have demonstrated that TCM offers significant advantages in alleviating pelvic pain and urinary symptoms by modulating multi-target networks [20]. Specifically, the integration of proteomics and metabolomics has revealed that herbal formulas can restore metabolic homeostasis and inhibit key inflammatory signaling pathways in the prostate microenvironment [21]. In this study, we integrated network pharmacology, metabolomics, and proteomics to systematically evaluate the therapeutic effects of BJT on EAP models and elucidate its underlying molecular mechanisms. In vitro experiments were further conducted to verify the expression of key regulatory proteins, thus identifying the core pathways mediating the pharmacological actions of this compound.

**Figure 1:**
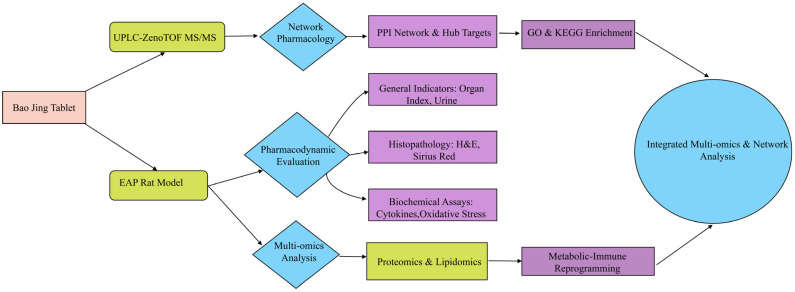
Overall experimental design and research workflow. This study integrates chemical profiling, network pharmacology, and in vivo experiments with multi-omics analysis to elucidate the therapeutic mechanism of BJT in CP/CPPS rats.

Notably, the potential role of metabolic reprogramming in BJT-mediated prostate protection remains unexplored. Emerging evidence suggests that HIF-1α-mediated glycolytic reprogramming plays a pivotal role in the functional transition of immune cells during chronic inflammatory disorders [22,23]. To address this gap, our integrated analysis revealed that BJT exerts its effects through multi-component synergistic actions, primarily by modulating relevant signal pathways to reverse glycolytic metabolic reprogramming within the local prostate microenvironment. This mechanism alleviates inflammatory responses, ameliorates tissue hypoxia and energy metabolism disorders, thereby achieving treatment of CP/CPPS at the metabolic-immune level (Figure 1). These findings elucidate the multi-target, multi-pathway regulatory mechanism of BJT’s therapeutic efficacy, providing both theoretical justification and experimental support for the use of traditional Chinese herbal formulas in treating chronic prostatitis.

## 2. Results

### 2.1. BJT Component Identification and Active Ingredient Prediction

**Figure 2:**
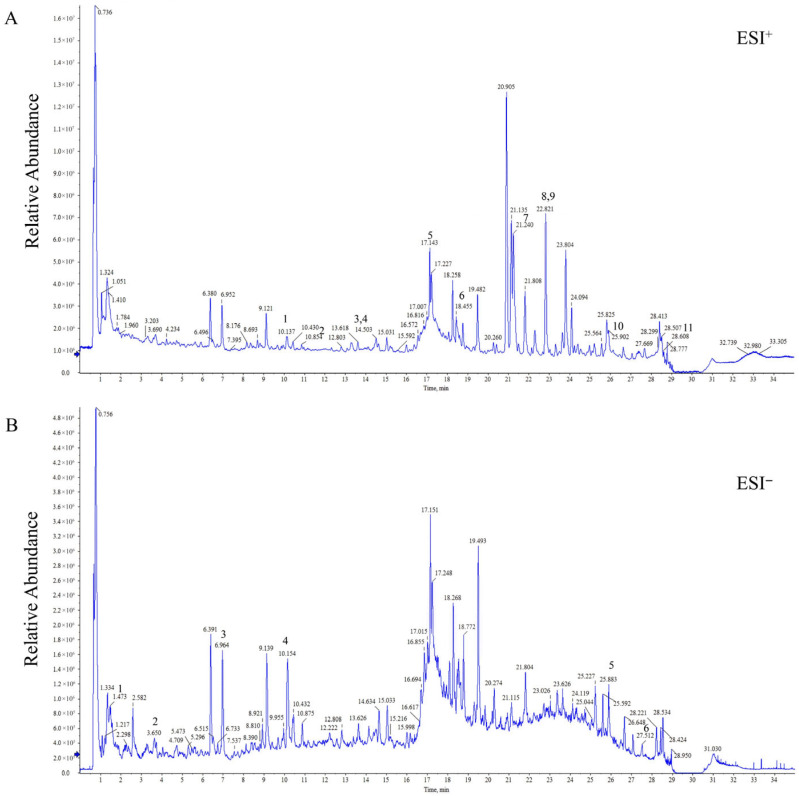
Representative UHPLC-MS base peak chromatograms (BPC) of BJT. (**A**) Base peak chromatogram in positive electrospray ionization (ESI+) mode. 1: Isoliquiritigenin; 2: (+)-syringaresinol; 3: Ononin; 4: Formononetin; 5: Yamogenin; 6: Curcumenol; 7: Schisandrin; 8: Bomyl acetate; 9: Curdione; 10: Germacrone; 11: Dehydropachymic acid; (**B**) Base peak chromatogram in negative electrospray ionization (ESI−) mode. 1: Adenine; 2: Caffeic acid; 3: 4′-Hydroxyacetophenone; 4: Liquiritin; 5: Poricoic acid A (F); 6: Naringenin. The peaks are well-separated within 35 min, demonstrating the multi-component chemical complexity of the BJT extract. The arrows in the figure indicate the retention times of the corresponding compounds.

To clarify the chemical composition of BJT, a comprehensive qualitative analysis of BJT’s chemical constituents was performed using UHPLC-Q-Orbitrap HRMS, identifying 174 compounds (Figure 2A,B). Following ADME (Absorption, Distribution, Metabolism, Excretion) screening principles, 54 major constituents were further selected, including kaempferol, formononetin, and emodin (Supplementary Table S1).

### 2.2. Network Pharmacology Predicts the Mechanism of BJT in Treating CP/CPPS

**Figure 3:**
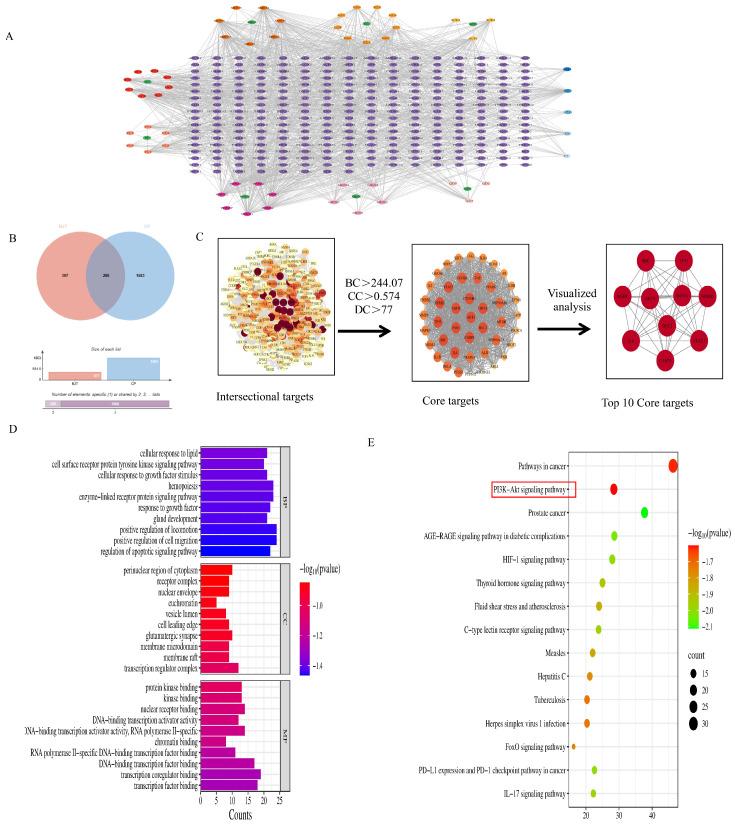
Network pharmacology-based identification of potential targets and therapeutic pathways of BJT against CP/CPPS. (**A**) Protein–protein interaction network of intersecting targets; (**B**) Venn diagram showing overlapping targets between BJT’s bioactive components and chronic prostatitis-related targets; (**C**) Interaction target network, with larger and redder nodes indicating higher functional relevance in the network; (**D**) Gene Ontology (GO) enrichment analysis, highlighting the top 10 significantly enriched terms in biological processes (BP), cellular components (CC), and molecular functions (MF). (**E**) KEGG pathway enrichment analysis, showing the top 15 enriched pathways; the size of the circles represents the gene count, and the color indicates the significance (*p*-value), with the PI3K/Akt and glycolysis pathways identified as pivotal signaling axes.

By predicting potential relationships among drug components, targets, and diseases, this approach helps researchers elucidate the therapeutic mechanisms of traditional Chinese medicinal formulas. To further clarify the mechanism of action of BJT in treating CP, we employed network pharmacology methods to predict potential intervention pathways. The Swiss-TargetPrediction database was utilized to predict 688 potential targets for these 54 constituents. Additionally, by querying the GeneCards, OMIM, TTD, and PharmGkb databases, we collected 1863 CP-related targets. Venn diagram analysis revealed overlapping targets between BJT and CP, identifying 280 common targets (Figure 3B). Furthermore, we constructed a BJT-active compound–CP target network comprising 341 nodes and 1535 edges (Figure 3A), indicating BJT exerts its therapeutic effect on CP through multi-component, multi-target mechanisms. Uploading the 280 common targets to the STRING database and subsequent analysis using Cytoscape 3.9.1 visualized the key targets. Topological analysis further identified 44 core targets, including Akt1, BCL2, HIF-1α, TP53, IL6, which may underpin BJT’s therapeutic efficacy against CP (Figure 3C). Metascape database analysis revealed the biological processes and molecular functions associated with these overlapping targets in hypothyroidism, identifying 1459 GO terms (1301 BP, 63 CC, and 95 MF). The top 10 most significant terms from each category were selected based on *p*-values and visualized (Figure 3D). KEGG pathway enrichment analysis identified 168 signaling pathways, with the top 15 enriched pathways displayed in a bubble plot (Figure 3E).

### 2.3. Protective Effects of BJT on Rats with EAP Model

**Figure 4:**
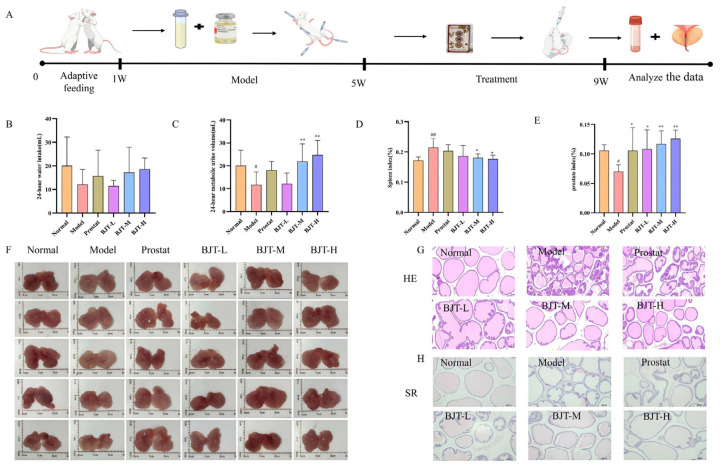
Therapeutic effects of BJT on general physiological indicators and prostatic histopathology in EAP rats. (**A**) Schematic experimental design illustrating the timeline for adaptive feeding, EAP modeling, BJT administration, and sample collection; (**B**) 24-h water intake (*n* = 7). Prostate tablets are commonly used medications in the current clinical treatment of prostate diseases. Therefore, we have set Prostate as the positive drug group; (**C**) 24-h metabolic urine volume (*n* = 7); (**D**) The spleen index of rats (*n* = 7); (**E**) The prostate index of rats (*n* = 7); (**F**) Gross morphology of the dissected prostate tissues across all groups, illustrating differences in volume, color, and texture; (**G**) Typical Hematoxylin and Eosin (H&E) staining of rat prostate (HE, 100×); (**H**) Representative Sirius Red staining (SR) of rat prostate (SR, 100×); Data are presented as mean ± standard deviation (SD). Differences between groups were analyzed by one-way analysis of variance (ANOVA) followed by Tukey’s post hoc test. # *p* < 0.05, ## *p* < 0.01, vs. normal group. * *p* < 0.05, ** *p* < 0.01, vs. model group.

To investigate the protective effects of BJT against prostatitis, we established an EAP model and administered the treatment for four weeks (Figure 4A: Modeling and Administration Cycle Diagram). Water intake and urine output serve as critical indicators for assessing rat prostatitis. Results revealed significantly reduced urine output in the model group, with only a slight decrease in water intake that did not reach statistical significance. Following administration, both BJT-Z and BJT-H groups exhibited significantly increased urine output, approaching normal levels, while water intake increased without reaching statistical significance (Figure 4B,C). Changes in spleen index reflect systemic immune status. The model group exhibited a significantly elevated spleen index (*p* < 0.01). Following administration, both BJT-Z and BJT-H groups demonstrated a significant reduction in spleen index (*p* < 0.01), suggesting that BJT may alleviate splenic immune burden by modulating immune responses (Figure 4D). In the model group, prostate tissue exhibited reduced volume and dull colouration. During dissection, it presented a hard texture with severe adhesions, making separation difficult. In contrast, the prostate volumes in the normal and treated groups were enlarged and showed vivid colouration. The tissue texture was moderately soft and easily separable during dissection. The prostate index in the model group was significantly reduced (*p* < 0.05). Meanwhile, the prostate index in the treated rats showed significant improvement (*p* < 0.05 or *p* < 0.01), consistent with histopathological findings (Figure 4E,F). The model group showed prostate histomorphological changes (Figure 4G,H). Compared with the normal group, rats in the model group exhibited marked lymphocytic infiltration between prostate glands, glandular structural damage, and glandular atrophy. Furthermore, Sirius Red staining (SR) revealed that the model group exhibited increased collagen deposition in the prostate compared to the normal group. The pathological status of the prostate tissue was improved after BJT intervention.

### 2.4. BJT Alleviates Oxidative Stress and Local Inflammation Against EAP-Induced Prostatitis

**Figure 5:**
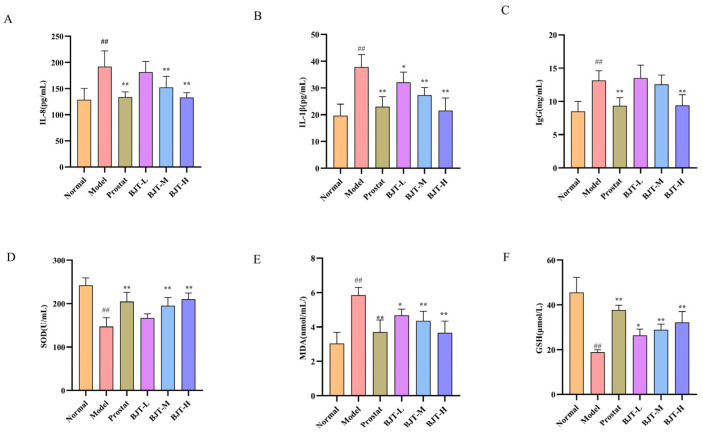
BJT alleviated oxidative stress and inflammatory response expression in EAP rats. (**A**–**C**) The expression levels of inflammatory factors IL-8, IL-1β, and IgG in each group (*n* = 7). (**D**–**F**) The expression levels of indicators of oxidative stress, SOD, MDA, and GSH in each group (*n* = 7). Data are presented as mean ± standard deviation (SD). Differences between groups were analyzed by one-way analysis of variance (ANOVA) followed by Tukey’s post hoc test. ## *p* < 0.01, vs. normal group. * *p* < 0.05, ** *p* < 0.01, vs. model group.

To evaluate the protective effect of BJT against EAP-induced prostatitis, serum levels of IgG, IL-8, and IL-1β were measured. Results demonstrated that compared with the model group, BJT-L, BJT-M, and BJT-H significantly reduced serum levels of IgG, IL-8, and IL-1β, with inhibitory effects increasing with dose (*p* < 0.01) (Figure 5A–C). Furthermore, to investigate the effects of BJT on oxidative stress in rat prostate tissue, this study measured serum levels of SOD, MDA, and GSH. Results indicated that, compared with the model group, increasing BJT doses significantly enhanced SOD and GSH activity while markedly reducing MDA levels (*p* < 0.01) (Figure 5D–F). In summary, BJT alleviates inflammatory responses and mitigates oxidative stress in chronic prostatitis.

### 2.5. Metabolomics Analysis of BJT on Rats with EAP Model 

**Figure 6:**
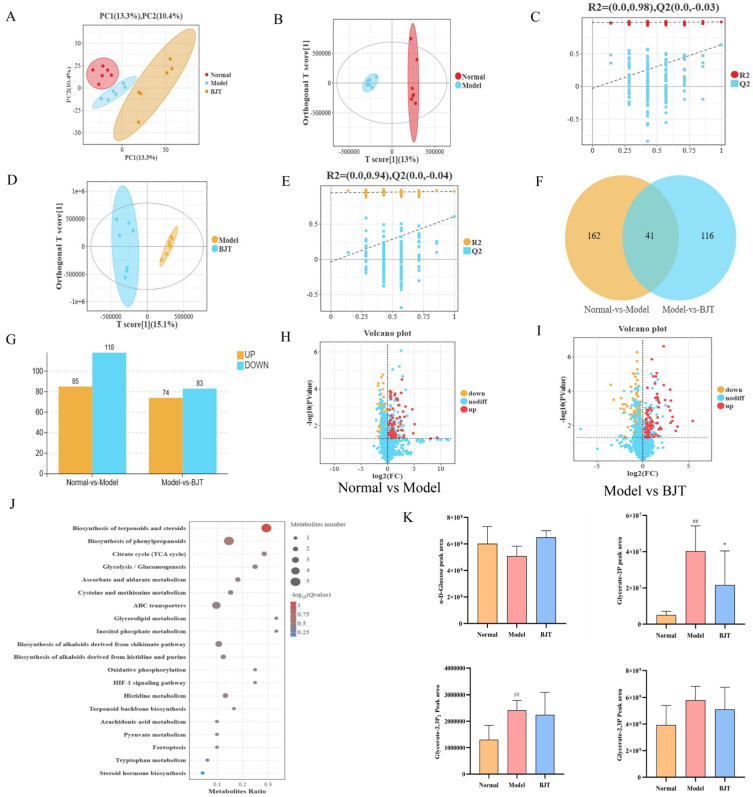
Metabolomics reveals differentially expressed metabolites in rats across experimental groups. (**A**) PCA score plot. (**B**,**C**) OPLS-DA Score Plot and Model Validation between the normal and the model group. (**D**,**E**) OPLS-DA Score Plot and Model Validation between the model and the BJT group. (**F**) Venn diagram of differentially expressed proteins overlapping between the normal vs. the model group and the model vs. the BJT group. (**G**–**I**) Different metabolite expression patterns among various groups. (**J**) KEGG metabolic pathway enrichment analysis. (**K**) Changes in differential metabolites. Data are presented as mean ± standard deviation (SD). Differences between groups were analyzed by one-way analysis of variance (ANOVA) followed by Tukey’s post hoc test. ## *p* < 0.01, vs. normal group. * *p* < 0.05, vs. model group.

To investigate the metabolic effects of BJT in the EAP rat model, non-targeted metabolomics analysis was performed on prostate tissue. Principal component analysis (PCA) revealed distinct separation between the normal and model groups, indicating successful modeling (Figure 6A). OPLS-DA further confirmed significant metabolic differences between these groups. Replacement tests yielded R2Y = 0.986, R2Y = 0.962, with no overfitting observed, validating the reliability of the OPLS-DA model (Figure 6B–E). Using thresholds of VIP > 1.0 and *p* < 0.05, 319 differentially expressed metabolites were identified, with an overlap of 41 differentially expressed metabolites (Figure 6F). Compared with the normal group, the model group exhibited 85 differentially upregulated metabolites and 118 differentially down-regulated metabolites. Compared with the model group, the BJT group exhibited 74 differentially upregulated metabolites and 83 differentially downregulated metabolites (Figure 6G–I). To further explore the potential mechanisms of BJT in treating prostatitis, pathway enrichment analysis was conducted on differentially expressed metabolites. KEGG analysis revealed multiple signaling and metabolic pathways, including biosynthesis of terpenoids and steroids, the citrate cycle, glycolysis, oxidative phosphorylation, arachidonic acid metabolism, and the HIF-1α signaling pathway (Figure 6J). These metabolic shifts highlight a state of metabolic reprogramming within the inflamed prostate. Specifically, the enrichment of the HIF-1α signaling pathway and glycolysis indicates that EAP induces a “Warburg-like” effect, where cells prioritize aerobic glycolysis over oxidative phosphorylation to sustain the inflammatory response and survive in a hypoxic microenvironment. Such metabolic alterations are known to drive the production of pro-inflammatory cytokines and exacerbate pelvic pain in CP/CPPS. Notably, key metabolites associated with glycolytic metabolism—including M179T285_NEG (α-D-Glucose), M333T352_2_POS (Glycerate-2,3P), M371T18_NEG (Glycerate-2P), and M87T113_NEG (Pyruvate)—were significantly elevated in the model group but notably reversed following BJT treatment (Figure 6K). This suggests that BJT exerts its therapeutic efficacy by rectifying this glycolytic flux, potentially leading to the suppression of the HIF-1α-mediated inflammatory cascade and the restoration of prostatic metabolic homeostasis.

### 2.6. Proteomics Analysis of BJT on Rats with EAP Model 

**Figure 7:**
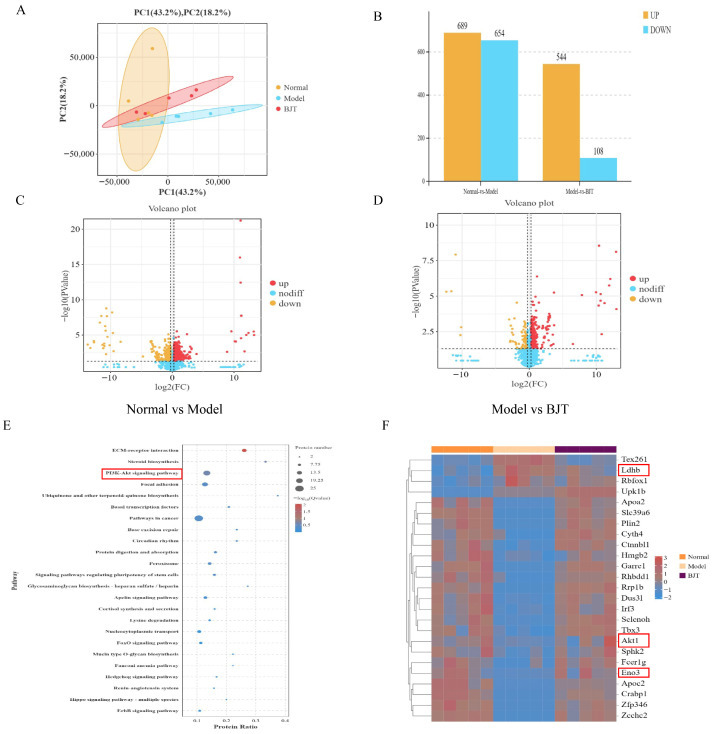
Proteomics reveals differentially expressed proteins in rats across experimental groups. (**A**) PCA diagram of principal component analysis. (**B**–**D**) Basic analysis of proteomics differences. (**E**) KEGG proteomics pathway enrichment analysis, with the PI3K/Akt signaling pathway identified as core therapeutic targets. (**F**) Heat map of differential proteins.

Proteomics techniques were employed to investigate the mechanism of BJT treatment in the EAP rat model. Principal Component Analysis (PCA) revealed significant differences between the normal, model, and BJT groups (Figure 7A). Differentially expressed proteins were screened using |log2FC| > 0.26 and *p* < 0.05, yielding 325 significantly differentially expressed proteins (Figure 7B). Compared to the normal group, the model group exhibited 689 upregulated proteins and 654 downregulated proteins (Figure 7C). Comparing the model group to the BJT group revealed 544 upregulated proteins and 108 downregulated proteins (Figure 7D). KEGG pathway enrichment analysis of differentially expressed proteins revealed significant enrichment in multiple pathways related to inflammation, cellular signaling, and metabolism. Most critically, the PI3K-Akt signaling pathway in the red box exhibited significant enrichment, directly corroborating the preliminary predictions from network pharmacology (Figure 7E). A heatmap of the top 20 differentially expressed proteins (based on *p*-value) identified several proteins occupying core regulatory nodes. Akt, a central kinase within the PI3K-Akt pathway, was identified as a differentially expressed protein. And its expression levels were significantly downregulated in the treatment group. Furthermore, we observed significant alterations in the expression of two key enzymes within the glycolysis pathway: lactate dehydrogenase B subunit (Ldhb) and enolase 3 (Eno3). These findings establish a direct link at the protein level between the inhibition of upstream signaling pathways and the regulation of downstream metabolic pathways (Figure 7F).

### 2.7. Validation of Key Proteins in the PI3K/Akt-HIF-1α-Glycolysis Signaling Axis

**Figure 8:**
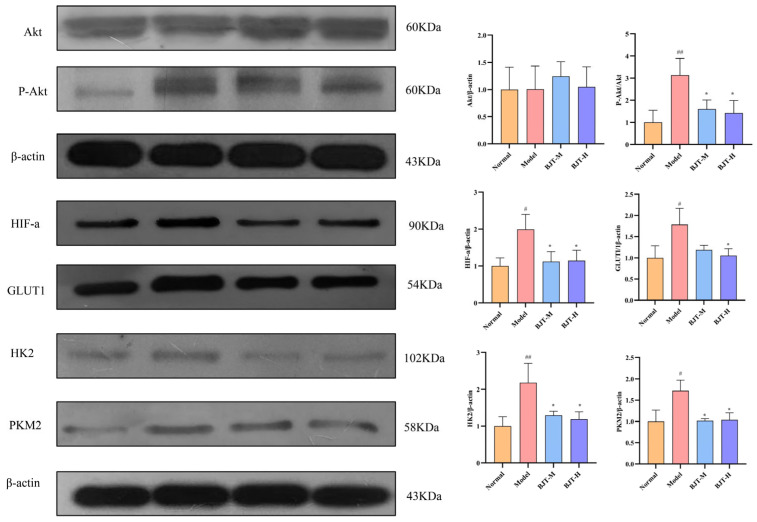
BJT modulates the PI3K/Akt-HIF-1α signaling axis and suppresses glycolytic enzymes in EAP rat prostate tissues. Expression of prostate tissue proteins: Akt, P-Akt (Ser473), GLUT1, HIF-1α, HK2, and PKM2 (*n* = 3). Data are expressed as mean ± standard deviation (SD). All data were subjected to normality tests. Multiple group comparisons were performed using one-way analysis of variance (ANOVA) followed by Tukey’s post hoc test for pairwise comparisons. # *p* < 0.05, ## *p* < 0.01, vs. normal group. * *p* < 0.05, vs. model group.

Through preliminary investigations, this study employed Western blot analysis to evaluate the regulatory effects of BJT on validating key proteins within the PI3K/Akt-HIF-1α-glycolysis signaling axis. The Western blot results showed that, compared to the normal group, the P-Akt, HIF-1α, GLUT1, HK2, and PKM2 expression levels in the model group were significantly higher (*p* < 0.01). Compared to the model group, the P-Akt, HIF-1α, GLUT1, HK2, and PKM2 expression levels in the BJT-M and BJT-H groups were significantly lower (*p* < 0.01) (Figure 8). These results suggest that BJT may exert a modulatory effect on the PI3K/Akt-HIF-1α-glycolysis axis, potentially by suppressing the overactivation of Akt and downstream glycolytic enzymes in the prostatic tissue of EAP rats.

## 3. Discussion

CP/CPPS is a common male condition characterized by high incidence, prolonged course, and tendency to recur, causing significant distress to sufferers and severely impacting their quality of life. Consequently, the exploration of more effective treatment approaches is imperative. Currently, the aetiology, pathogenesis, and pathophysiology of CP/CPPS remain incompletely understood. However, mounting evidence suggests that inflammatory factors, oxidative stress theories, urinary dysfunction, immune dysregulation, and metabolic disorders may interact during disease progression, collectively influencing CP/CPPS pathogenesis. The precise mechanisms warrant further investigation [24,25,26]. Research indicates that BJT therapy for chronic prostatitis derives its efficacy from synthesizing the theoretical insights of numerous specialists. It specifically addresses the pathogenesis involving deficiency of the spleen and kidney, combined with internal obstruction by damp-heat and blood stasis. The treatment approach centers on “tonifying the kidney to consolidate essence and draining dampness to eliminate turbidity” to cure chronic prostatitis [9,27].

With our increasing understanding of the pathogenesis of chronic prostatitis, autoimmune mechanisms have garnered greater attention. Consequently, establishing a reliable autoimmune prostatitis model has become a focal point of research. In the EAP model, prostatic steroid-binding protein serves as the primary autoantigen, capable of eliciting both cellular and humoral immune responses. However, not all rodents are suitable for autoimmune modeling. Consequently, this study employs Wistar rats as the EAP model, selected for their pathological features resembling those of human chronic prostatitis [28]. Prostat tablets are commonly used drugs in the current clinical treatment of prostate diseases. They are widely used in the treatment of prostate diseases such as benign prostatic hyperplasia and chronic prostatitis [29]. Its mechanism of action is clear, mainly comprising anti-androgenic, anti-inflammatory, and anti-immunomodulatory effects [30,31]. Therefore, it is selected as the positive control drug.

Inflammatory mediators, as pivotal immune regulatory proteins, play a crucial role in mediating physiological inflammatory responses and maintaining immune homeostasis [32]. Pro-inflammatory mediators such as IL-1β and IL-8 are considered proximal risk factors for CP/CPPS. The primary symptoms of CP/CPPS include pain, urinary abnormalities, and altered sexual function, with pain being the principal factor affecting patients’ quality of life and driving their clinical presentations. Previous studies have confirmed that abnormal expression of IL-1β, IL-8, and their receptors can directly influence the progression of pain [33,34]. IgM and IgG are primary effector molecules in systemic humoral immune responses, with IgG predominantly synthesized by splenic and lymph node plasma cells. Due to its high concentration and strong penetrating power, IgG plays a widespread role in bodily defense [35,36]. In chronic prostatitis, abnormal elevation of IgG typically indicates the presence of “autoimmune abnormalities” [37]. In the study, ELISA results demonstrated significantly elevated levels of pro-inflammatory cytokines IL-1β, IL-8, and IgG in model serum compared to the normal group. Following treatment with varying doses of BJT, expression of IL-1β, IL-8, and IgG decreased. This indicates that BJT may treat CP/CPPS by reducing inflammatory mediators.

The aetiology and pathogenesis of CP/CPPS remain incompletely understood, though oxidative stress has been demonstrated to be closely associated with the condition [38,39]. Oxidative stress disrupts the body’s metabolism of oxygen radicals, leading to excessive free radical production and diminished scavenging capacity. This results in lipid peroxidation damage to cellular proteins, DNA, cell membranes, and cytoplasm [40]. SOD and GSH are endogenous antioxidant enzymes that convert superoxide into hydrogen peroxide, which is subsequently converted into water, reflecting the body’s antioxidant capacity. MDA is a marker of oxidative damage and the final product of lipid peroxidation. These molecules indicate both oxidative stress-induced injury and antioxidant defense capabilities [41]. In the study, CP/CPPS induced by the EAP model was associated with reduced activity of antioxidant enzymes. BJT alleviated oxidative stress damage in CP/CPPS by decreasing MDA levels and increasing SOD and GSH activity.

**Figure 9:**
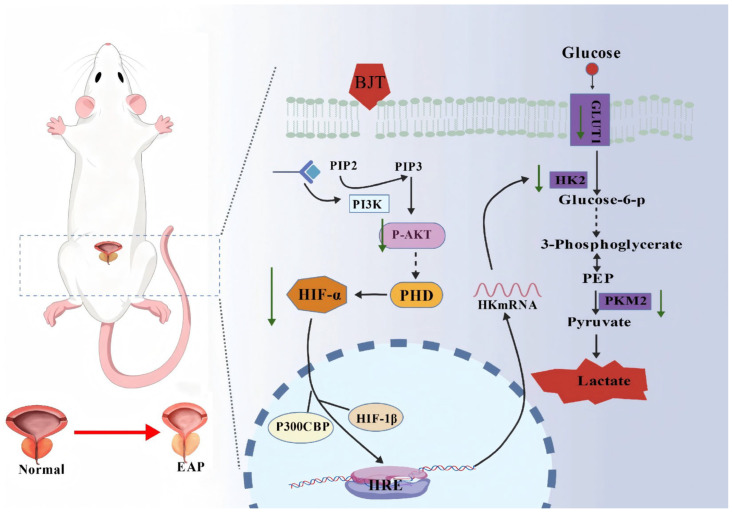
Mechanisms of action of BJT for chronic prostatitis therapy. The green arrows in the figure indicate the decrease in protein expression after treatment with BJT.

The integration of network pharmacology, metabolomics, and proteomics has emerged as a potent approach for investigating the therapeutic effects of multi-component traditional Chinese herbal medicines [42]. Network pharmacology analysis suggests that BJT may treat CP/CPPS by targeting the PI3K/Akt signaling pathway, while both metabolomics and proteomics indicate significant alterations in glycolysis-related proteins. The PI3K/Akt signaling pathway maintains intricate connections with glycolysis, with its interactions playing a pivotal role in cellular metabolism, growth, and survival. The PI3K/Akt pathway regulates glucose transporters and glycolysis through direct post-translational modifications. Furthermore, it influences downstream transcription factors to ensure sustained regulation [43,44]. For instance, Akt increases mitochondrial HK2 content by mediating its phosphorylation at Thr-473, thereby preventing its mitochondrial disassociation [45]. Concurrently, Akt reduces HIF-1α hydroxylation by modulating prolyl hydroxylases (PHDs), thereby elevating HIF-1α protein expression [46]. Moreover, HIF1-α not only induces the expression of key glycolytic enzymes, such as GLUT1, but also upregulates HK2 and PKM2 [47]. Collectively, these findings demonstrate that HIF-1α is intrinsically linked to the glycolytic pathway, influencing cellular energy supply under hypoxic conditions [48,49]. Our findings indicate that following BJT treatment for CP/CPPS, P-Akt levels are downregulated. Concurrently, glycolytic capacity, including glucose uptake and lactate production, is significantly inhibited, alongside reduced expression of glycolytic enzymes such as GLUT1, HK2, and PKM2. This demonstrates that BJT can treat CP/CPPS by modulating the PI3K/Akt signaling pathway and suppressing the expression of glycolytic enzymes (Figure 9).

The novelty of this study lies in the transition from traditional symptom-based evaluation to a deep understanding of metabolic-immune crosstalk in CP/CPPS. By integrating multi-omics data, we demonstrated, for the first time, that the anti-inflammatory effect of BJT is coupled with metabolic reprogramming of the prostate microenvironment. Specifically, modulation of the PI3K/Akt-HIF-1α-glycolysis axis represents a previously unrecognized mechanism by which Chinese patent medicines treat chronic prostatitis. These findings not only clarify the multi-target essence of BJT but also suggest that targeting ‘metabolic checkpoints’ could be a promising therapeutic strategy for managing chronic pelvic pain and inflammatory damage.

Despite the multi-omics evidence, the current study lacks direct functional validation using specific inhibitors or gene-silencing techniques. Furthermore, although BJT reduced key pro-inflammatory pain mediators, direct behavioral assessments of pelvic pain were not performed. Future studies are warranted to further strengthen the causal links and evaluate the analgesic effects of BJT.

In conclusion, BJT alleviates local inflammation and oxidative stress, modulates the PI3K/Akt-HIF-1α-glycolysis signaling axis, and improves chronic prostatitis from an immunometabolic perspective. This provides novel insights for treating CP/CPPS and offers evidence for future clinical applications of BJT.

## 4. Materials and Methods

### 4.1. Reagents and Materials

Interleukin-8 (IL-8) (20240318I4), Interleukin-1β (IL-1β) (2240318R5), and Immunoglobulin G(IgG) (2020317G1) were purchased from Jiangsu Baolai Biotechnology Co., Ltd. (Nanjing, China). Reduced glutathione (GSH) (A006-2-1) and superoxide dismutase (SOD) (A001-3-2) were purchased from Nanjing Jiancheng Bioengineering Institute (Nanjing, China). Antibodies against the following targets were purchased: Akt (A18120) from Abclonal Technology Co., Ltd. (Wuhan, China). P-Akt, (80455-1-RR) and β-actin(81115-1-RR) were purchased from Proteintech Group (Wuhan, China). HK2 (TD6176F), GLUT1 (T55360F), PKM2 (MH68072F) were purchased from Abmart Inc. (Shanghai, China). HIF-1α(AF1009) was purchased from Affinity Biosciences (Jiangsu, China). Additional reagents were purchased: Bao Jing Tablets (BJT) (produced by Jiangsu Provincial Hospital of Traditional Chinese Medicine, approval number: Su Zhi Yao Zi Z04000524, Nanjing, China); Prostat Tablets (Meirui Pharmaceutical, 23040500, Nanjing, China); Physiological Saline (Kelun Pharmaceutical, N23011308, Chengdu, China); 0.1 M PBS Buffer (Solarbio, 20230304, Beijing, China); Complete Freund’s Adjuvant (Sigma, 0000248755, St. Louis, MO, USA); Malondialdehyde (MDA) (Aidisheng, ADS-W-Y-H002, Jiangsu, China). 

### 4.2. Preparation and Component Analysis of Bao Jing Tablets

The BJT powder was ground in 70% methanol, homogenized, and centrifuged. Chromatographic separation was performed on a Waters Acquity UPLC I-Class system equipped with a Waters CORTECS UPLC T3 column (2.1 × 100 mm, 1.6 µm) maintained at 40 °C. Gradient elution was carried out using 0.1% formic acid in water (mobile phase A) and acetonitrile (mobile phase B) at a flow rate of 1 mL/min. The injection volume was 10 μL, and the detection wavelength was set at 250 nm. High-resolution mass spectrometry was conducted on an AB SCIEX ZenoTOF 7600 system (SCIEX, Framingham, MA, USA). Data were acquired in both positive and negative ion modes over the *m*/*z* range of 100–1500. In the positive-ion mode, the spray voltage was 5500 V, and the capillary temperature was 300 °C. In the negative-ion mode, the spray voltage was set to 4500 V.

From the analysis results, compounds with mzCloud composite scores above 70 were further screened using the Traditional Chinese Medicine Systems Pharmacology Database (TCMSP). And then, network pharmacology analysis was performed on these components.

### 4.3. Network Pharmacology Construction

Network pharmacology analysis was performed on components identified by UPLC-ZenoTOF 7600-MS/MS. Compounds were screened from the TCMSP [50] based on ADME (Absorption, Distribution, Metabolism, and Excretion) properties, specifically requiring oral bioavailability (OB) ≥ 30% and drug-likeness (DL) ≥ 0.18. The SwissADME database was simultaneously employed to cross-validate compounds, selecting those meeting the criteria ≥ 2 “YES” for “Druglike” (Lipinski, Ghose, Veber, Egan, Muegge) and high GI absorption [51]. All compounds screened via the aforementioned methods were consolidated. For target prediction, the canonical SMILES strings of the screened active ingredients were obtained from the PubChem database. These strings were then imported into the SwissTargetPrediction database, setting the species to “Homo sapiens” and selecting targets with a probability score > 0. Target information for Bao Jing tablets was sourced from GeneCards (using a Relevance score ≥ 1.0 as the threshold), Disgenet, and OMIM. Disease targets from these three databases were then merged, duplicates removed, and standardized using UniProt. Overlapping genes between Bao Jing tablets and chronic prostatitis were identified using Venn diagrams. Protein–protein interaction (PPI) networks were constructed using the STRING database with a required minimum combined confidence score of 0.4. Visualization was performed in Cytoscape 3.9.1, and the core targets were identified using topological analysis based on Degree, Betweenness, and Closeness centrality metrics. The Metascape database was employed for GO and KEGG pathway enrichment analyses, with *p*-values < 0.01 considered statistically significant.

### 4.4. Animal Grouping and Administration

Forty-two male SPF-grade Wistar rats were procured from Speifu (Suzhou, China) Biotechnology Co., Ltd. (License No.: SCXK (Su)2022-0006). The animals were housed in the SPF-grade animal laboratory of Jiangsu Suzhong Pharmaceutical Group Biopharmaceutical Co., Ltd. (Taizhou, China). The experimental animal permit number is SYXK (Su) 2022-0057. The rats were acclimated to the environment for one week before the start of the experiment, provided with regular pellet feed, and had free access to food and water. Room temperature was maintained at 20–24 °C, relative humidity at 45–65%, and a 12-h light-dark cycle. The experiment was approved by the Animal Ethics Committee of Jiangsu Suzhong Biopharmaceutical Co., Ltd. (Approval No. 2023112001).

An experimental autoimmune prostatitis (EAP) model was employed [24,52]. Fresh prostate tissue was homogenized in 0.5% Triton X-100 saline (Sigma Aldrich, St. Louis, MO, USA). The supernatant was centrifuged to obtain a purified extract. Concentration was measured using the bicurefaction method, then diluted to 60 mg·mL^−1^ in 0.01 mol·L^−1^ PBS. This was mixed in equal volumes with complete Freund’s adjuvant for subsequent use. Following 7 days of acclimatization feeding, the normal group (7 rats) received subcutaneous saline injections. The remaining 35 rats underwent modeling via five-point subcutaneous injection of the emulsion on days 1 and 28 (0.4 mL dorsally on the neck, 0.2 mL bilaterally in the inguinal regions, and 0.1 mL bilaterally on the hindfoot pads). Following modeling, rats were randomly assigned to the model group, the low-dose BJT group (BJT-L), the medium-dose BJT group (BJT-M), the high-dose BJT group (BJT-H), and the positive drug group for oral administration over 4 weeks. The blank and model groups received saline solution. The BJT-L, BJT-M, and BJT-H groups were administered by gavage at dosages of 197.2 mg·kg^−1^, 591.5 mg·kg^−1^, and 1774.5 mg·kg^−1^, respectively, corresponding to 1/3, 1, and 3 times the clinically equivalent dose for humans. The positive drug group received 34.2 mg·kg^−1^ Puxutai tablets solution.

### 4.5. Sample Collection

Following administration, rats were placed in metabolic cages and fasted without water restriction for a 24-h urine collection. The following day, after weighing, rats were anesthetized, blood was collected from the abdominal aorta, and the supernatant was stored at −80 °C. Prostate and spleen tissues were dissected, weighed, and organ coefficients calculated as tissue weight relative to body weight. A portion of prostate tissue was rapidly frozen in liquid nitrogen and stored at −80 °C, and the remainder was immersed in 10% formalin for fixation.

### 4.6. Histopathological Analysis

The fixed prostate tissue was paraffin-embedded, sectioned, and stained with Hematoxylin–Eosin staining and Sirius Red staining. The pathological state of rat prostate tissue was observed under an optical microscope and photographed to assess prostate tissue damage and inflammatory conditions.

### 4.7. Enzyme-Linked Immunosorbent Assay

Frozen rat serum was retrieved and processed according to the ELISA kit protocol to determine levels of IL-8, IL-1β, IgG, SOD, MDA, and GSH. An enzyme-linked immunosorbent assay measured absorbance values, which were then converted into corresponding cytokine concentrations.

### 4.8. Metabolomics

Thawed serum samples were mixed with pre-chilled extraction buffer, followed by protein precipitation and centrifugation at 14,000× *g* for 20 min at 4 °C. The supernatant was then dried. Resuspend the dried residue in solvent, centrifuge, and collect the supernatant for LC-MS analysis. Chromatographic separation was employed on an ACQUITY UPLC BEH Amide column (2.1 mm × 100 mm, 1.7 μm), maintained at 25 °C with a flow rate of 0.5 mL/min, and a 2 μL injection volume. The mobile phase A consisted of 25 mM ammonium acetate and 25 mM ammonium hydroxide in water, while phase B was acetonitrile. The gradient elution was set as follows: 0–0.5 min, 95% B; 0.5–7 min, 95–65% B; 7–8 min, 65–40% B; 8–9 min, 40% B; 9–9.1 min, 40–95% B; 9.1–12 min, 95% B. The samples were maintained at 4 °C in the autosampler. Mass spectrometry acquisition employed both positive and negative ion modes on a ZenoTOF 7600 system (AB Sciex), with an ion spray voltage floating (ISVF) of ±5500 V. The source temperature was 500 °C, and the declustering potential (DP) was 80 V. The primary scan range was *m*/*z* 60–1000 Da, with secondary scanning at *m*/*z* 25–1000 Da. Data were acquired in information-dependent acquisition (IDA) mode with collision energy (35 ± 15) eV. Raw data were converted to MzML format using ProteoWizard 3.0.6428, followed by peak alignment, retention time correction, and peak area extraction via XCMS. To ensure data quality, metabolites with missing values exceeding 50% in all groups were filtered and excluded. The remaining missing values were imputed using the K-nearest neighbors (KNN) algorithm. Subsequently, the data were normalized by the total peak area and log-transformed to achieve a normal distribution. For multivariate statistical analysis, the data were further subjected to Auto-scaling (mean-centering and scaling to unit variance) to ensure each metabolite contributed equally to the model. Metabolites with missing values exceeding 50% were excluded, and remaining missing values were imputed using KNN and normalized by total peak area. PCA was utilized as an unsupervised method to visualize the overall distribution of samples, assess experimental reproducibility, and identify potential outliers. Principal Component Analysis (PCA), Orthogonal Partial Least Squares Discriminant Analysis (OPLS-DA), differential expression analysis, Gene Ontology (GO) annotation, and Kyoto Encyclopedia of Genes and Genomes (KEGG) pathway enrichment analysis were performed using the Omicsmart (www.omicshare.com/tools/, accessed on 23 June 2025). Differentially expressed metabolites were identified using VIP > 1.0 and *p* < 0.05.

### 4.9. Proteomics

Frozen tissue samples were processed by adding pre-chilled 8 M urea and 1% SDS lysis buffer (containing protease inhibitors) in proportion. Following tissue homogenisation using a tissue grinder and ice-cold lysis, the supernatant was collected by centrifugation. Subsequently, the iST Sample Preparation Kit was employed for protein denaturation, trypsin digestion, and peptide desalting. The resulting peptides were stored frozen for subsequent use. Before mass spectrometry analysis, peptides were resuspended in 0.1% formic acid aqueous solution. Data were acquired via a 60-min gradient LC-MS/MS separation on an EASY-nLC 1200 system coupled to the mass spectrometer. The flow rate was 300 nL/min, and the mobile phase B (80% acetonitrile with 0.1% formic acid) increased from 5% to 35% over 45 min, followed by a rise to 100% for 5 min. Spectronaut 18 software analyzed the DIA data, with retention times calibrated using the iRT Kit. Principal component analysis (PCA), Gene Ontology (GO) annotation, and Kyoto Encyclopedia of Genes and Genomes (KEGG) pathway enrichment analysis were performed using the Omicsmart (www.omicshare.com/tools/, accessed on 23 June 2025). Differentially expressed proteins were identified using the criterion |log_2_FC| > 0.26 (fold change > 1.2-fold) and *p* < 0.05.

### 4.10. Western Blotting

Rat prostate tissues were lysed in RIPA lysis buffer (Aidisheng, Jiangsu, China) supplemented with protease inhibitors (Aidisheng, Jiangsu, China) and phosphatase inhibitors (Aidisheng, Jiangsu, China). The supernatant was collected after centrifugation at 12,000× *g* for 15 min at 4 °C. Protein concentration was determined using a BCA Protein Assay Kit (Aidisheng, Jiangsu, China). A total of 40 μg of the protein sample was loaded into sodium dodecyl sulfate polyacrylamide gel electrophoresis (SDS-PAGE). After being transferred to a PVDF membrane and blocked with 5% non-fat milk for 2 h at room temperature, the membrane was incubated with the primary antibody, then with the secondary antibody. Peroxidase activity in the bands was detected using a chemiluminescent detection kit (Vazyme, Jiangsu, China). The primary and secondary antibodies used were: P-Akt (1:1000; Proteintech), Akt (1:1000; Abclonal), PKM2 (1:1000; Abmart), HK2 (1:1000; Abmart), HIF-1α (1:1000; Affinity), GLUT1 (1:1000; Abmart), and β-actin (1:3000; Proteintech). Relative protein expression levels were calculated as the ratio of target protein band intensity to β-Actin intensity.

### 4.11. Statistical Analysis

All data were presented as means ± standard deviation (SD). Data were analyzed using GraphPad Prism 8.0. Statistical significance between groups was determined by one-way analysis of variance (ANOVA) followed by Tukey’s post hoc test for multiple comparisons. A *p*-value < 0.05 was considered statistically significant.

## 5. Conclusions

In this study, we demonstrated that BJT attenuates prostatic inflammation and pathological damage in an EAP-induced rat model of chronic pain/chronic prostatitis/chronic pelvic pain syndrome (CP/CPPS) by reducing inflammatory cytokine levels, improving oxidative stress status, and modulating apoptosis. Integrated metabolomics and network pharmacology studies revealed that BJT may regulate the PI3K/Akt–HIF-1α signaling pathway and glycolysis in CP/CPPS, findings validated by Western blot analysis. This multi-omics approach elucidates the potential mechanism of BJT in alleviating the biological drivers of CP/CPPS, with insights providing robust evidence for future clinical applications. Collectively, BJT shows promise as an effective therapeutic agent for mitigating the inflammatory and metabolic dysregulation associated with CP/CPPS.

## Data Availability

The data reported in this paper have been deposited in the OMIX, China National Center for Bioinformation/Beijing Institute of Genomics, Chinese Academy of Sciences (https://ngdc.cncb.ac.cn/omix, accessed on 24 January 2026: accession no. OMIX014604 and accession no. OMIX014614).

## Supplementary Materials

The following supporting information can be downloaded at https://www.mdpi.com/article/10.3390/ph19040632/s1. Table S1. LC/MS information of bioactive components in BJT.

## Abbreviations

BJT	Bao Jing Tablet
CP/CPPS	chronic prostatitis/chronic pelvic pain syndrome
EAP	experimental autoimmune prostatitis
TCM	Traditional Chinese Medicine
ADME	Absorption, Distribution, Metabolism, Excretion screening principles
PPI	protein–protein interaction
GO	Gene Ontology
BP	biological processes
CC	cellular components
MF	molecular functions
KEGG	Kyoto Encyclopedia of Genes and Genomes
SR	Sirius Red staining
H&E	Hematoxylin and Eosin staining
SD	standard deviation
ANOVA	analyzed by one-way analysis of variance
PCA	principal component analysis
PHDs	prolyl hydroxylases
IL-8	interleukin-8
IL-1β	interleukin-1β
IgG	immunoglobulin G
GSH	reduced glutathione
SOD	superoxide dismutase
MDA	malondialdehyde
Akt	protein kinase B
Akt	phosphorylated Akt
HK2	hexokinase 2
GLUT1	glucose transporter 1
PKM2	pyruvate kinase M2
HIF-1α	hypoxia-inducible factor-1α
β-actin	beta-actin
TCMSP	Traditional Chinese Medicine Systems Pharmacology Database
OB	oral bioavailability
DL	drug-likeness
OPLS-DA	Orthogonal Partial Least Squares Discriminant Analysis
SDS-PAGE	sodium dodecyl sulfate polyacrylamide gel electrophoresis
