# Supplementary material for: Mechanism of Bao Jing Tablets in Chronic Prostatitis/Chronic Pelvic Pain Syndrome: Insights from Multi-Omics and Network Pharmacology
Source: Pharmaceuticals (Basel). 2026 Apr 17;19(4):632. doi: 10.3390/ph19040632 (PMC13118977; doi:10.3390/ph19040632)
Supplement: Supplementary file 1 [file pharmaceuticals-19-00632-s001.zip › pharmaceuticals-4174287-supplementary.pdf]

Table S1 LC/MS information of bioactive components in BJT

No.	Compound	RT/min	Adduction	m/z	Source	identification level
1	Schisantherin C	24.85	[M+Na]+	554.2397	Schisandrae Chinensis Fructus	fragmentation
2	Schisandrin	21.23	[M+H]+	433.2222	Schisandrae Chinensis Fructus	fragmentation
3	Schisandrin C	27.85	[M+H]+	385.1646	Schisandrae Chinensis Fructus	fragmentation
4	Schizandrol B	22.27	[M+H]+	417.1907	Schisandrae Chinensis Fructus	fragmentation
5	Schisandrin A	26.7	[M+H]+	417.2274	Schisandrae Chinensis Fructus	fragmentation
6	Gomisin D	21.9	[M+H]+	531.2227	Schisandrae Chinensis Fructus	fragmentation
7	Schisanhenol	25.19	[M+H]+	403.2113	Schisandrae Chinensis Fructus	fragmentation
8	Ononin	14.45	[M+H]+	431.1336	Cuscutae Semen, licorice	fragmentation
9	Kaempferol	12.41	[M+H]+	287.0553	Cuscutae Semen, licorice, Verbenae Herb	fragmentation
10	Pinoresinol	12.09	[M+H]+	341.1383	Cuscutae Semen,Schisandrae Chinensis Fructus	fragmentation
11	alpha-Asarone	23.26	[M+H]+	209.117	Acoritataninowii Rhizoma	fragmentation
12	Emodin	22.65	[M+H]+	271.0597	Acoritataninowii Rhizoma	fragmentation
13	5-Hydroxymethylfurfural	2.22	[M+H]+	127.0389	Acoritataninowii Rhizoma	fragmentation
14	Bomyl acetate	22.82	[M+H]+	137.132	Acoritataninowii Rhizoma	fragmentation
15	Indirubin	22.5	[M+H]+	263.0812	Indigo Naturalis	fragmentation
16	Indigo Dye	20.83	[M+H]+	263.0815	Indigo Naturalis	fragmentation
17	(+)-syringaresinol	12.78	[M+H]+	401.1595	Indigo Naturalis	fragmentation
18	Formononetin	14.45	[M+H]+	269.0809	Dioscoreae Septemlo Bae Rhizoma,licorice	fragmentation
19	4'-Hydroxyacetophenone	6.92	[M-H]-	135.0446	Dioscoreae Septemlo Bae Rhizoma	fragmentation
20	Hederagenin	24.49	[M-H]-	471.3471	Dioscoreae Septemlo Bae Rhizoma	fragmentation
21	Columbin	21.42	[M-H]-	313.2375	Dioscoreae Septemlo Bae Rhizoma	fragmentation
22	Yamogenin	17.15	[M+H]+	415.3205	Dioscoreae Septemlo Bae Rhizoma	fragmentation
23	Diosgenin	22.26	[M+H]+	415.3204	Dioscoreae Septemlo Bae Rhizoma	fragmentation
24	Protocatechuic Aldehyde	3.85	[M-H]-	137.0243	Verbenae Herb	fragmentation
25	Caffeic acid	3.64	[M-H]-	179.0348	Verbenae Herb	fragmentation
26	Pectolinarigenin	21.51	[M-H]-	313.0709	Verbenae Herb	fragmentation
27	Isorhamnetin	22.19	[M-H]-	315.2533	Verbenae Herb	fragmentation

No.	Compound	RT/min	Adduction	m/z	Source	identification level
28	4-Hydroxybenzoic acid	2.26	[M-H]-	137.0241	Verbenae Herb	fragmentation
29	Diosmetin	17.78	[M+FA-H]-	299.0551	Verbenae Herb	fragmentation
30	Apigenin	12.81	[M+H]+	271.0601	Verbenae Herb	fragmentation
31	Protocatechuic acid	3.26	[M-H]-	153.0554	licorice,Acoritataninowii Rhizoma	fragmentation
32	Liquiritin	10.16	[M-H]-	417.119	licorice	fragmentation
33	Vanillic acid	5.27	[M+FA-H]-	167.0344	licorice	fragmentation
34	Isoliquiritigenin	10.14	[M+H]+	257.0809	licorice	fragmentation
35	p-Coumaric acid	7.73	[M-H]-	163.0396	licorice	fragmentation
36	Adenine	1.45	[M-H]-	134.0469	licorice	fragmentation
37	Naringenin	27.52	[M-H]-	271.2271	licorice	fragmentation
38	Glycyrrhetinic acid	26.6	[M+H]+	471.3473	licorice	fragmentation
39	Glabridin	23.19	[M+H]+	325.1433	licorice	fragmentation
40	16alpha-Hydroxydehydrotrametenolic acid	24.71	[M+H]+	453.3363	Poria Cocos(Schw.) Wolf.	fragmentation
41	Dehydropachymic acid	28.61	[M+H]+	509.3625	Poria Cocos(Schw.) Wolf.	fragmentation
42	Pachymic acid	28.78	[M-H]-	527.3731	Poria Cocos(Schw.) Wolf.	fragmentation
43	Poricoic acid A(F)	25.84	[M-H]-	497.3266	Poria Cocos(Schw.) Wolf.	fragmentation
44	4-hydroxybenzalde	5.47	[M-H]-	121.0293	Poria Cocos(Schw.) Wolf.	fragmentation
45	Poricoic acid B	25.09	[M-H]-	483.3113	Poria Cocos(Schw.) Wolf.	fragmentation
46	Isoferulic acid	4.72	[M-H2O-H]-	193.0502	Curcumae Rhizoma	fragmentation
47	4-Hydroxycoumarin	0.67	[M-H]-	160.9761	Curcumae Rhizoma	fragmentation
48	Curcumin	22.27	[M+H]+	369.1693	Curcumae Rhizoma	fragmentation
49	Curcumol	25.72	[M+H]+	237.1849	Curcumae Rhizoma	fragmentation
50	Curcumenol	18.5	[M+H]+	2350 1693	Curcumae Rhizoma	fragmentation
51	Curdione	22.82	[M+H]+	237.185	Curcumae Rhizoma	fragmentation
52	Germacrone	25.91	[M+H]+	219.1744	Curcumae Rhizoma	fragmentation
53	Angelicin	7.8	[M+H]+	187.0866	Curcumae Rhizoma	fragmentation
54	Bisdemethoxycurcumin	16.85	[M+H]+	309.0969	Curcumae Rhizoma	fragmentation
